# Cognitive load assessment through EEG: A dataset from arithmetic and Stroop tasks

**DOI:** 10.1016/j.dib.2025.111477

**Published:** 2025-03-19

**Authors:** Ali Nirabi, Faridah Abd Rahman, Mohamed Hadi Habaebi, Khairul Azami Sidek, Siti Yusoff

**Affiliations:** Department of Electrical and Computer Engineering, University Islam Antarabangsa, Jalan Gombak, Selangor, Malaysia

**Keywords:** EEG signals, Mental stress, Stress detection dataset, Artificial intelligence, Deep learning algorithms

## Abstract

This study introduces a thoughtfully curated dataset comprising electroencephalogram (EEG) recordings designed to unravel mental stress patterns through the perspective of cognitive load. The dataset incorporates EEG signals obtained from 15 subjects, with a gender distribution of 8 females and 7 males, and a mean age of 21.5 years [1]. Recordings were collected during the subjects' engagement in diverse tasks, including the Stroop color-word test and arithmetic problem-solving tasks. The recordings are categorized into four classes representing varying levels of induced mental stress: normal, low, mid, and high. Each task was performed for a duration of 10–20 s, and three trials were conducted for comprehensive data collection. Employing an OpenBCI device with an 8-channel Cyton board, the EEG captures intricate responses of the frontal lobe to cognitive challenges posed by the Stroop and Arithmetic Tests, recorded at a sampling rate of 250 Hz. The proposed dataset serves as a valuable resource for advancing research in the realm of brain-computer interfaces and offers insights into identifying EEG patterns associated with stress.

The proposed dataset serves as a valuable resource for researchers, offering insights into identifying EEG patterns that correlate with different stress states. By providing a solid foundation for the development of algorithms capable of detecting and classifying stress levels, the dataset supports innovations in non-invasive monitoring tools and contributes to personalized healthcare solutions that can adapt to the cognitive states of users. This study's foundation is crucial for advancing stress classification research, with significant implications for cognitive function and well-being.

Specifications Table

**Table:** 

Subject	Signal Processing
Specific subject area	EEG signal
Type of data	Raw
Data source location	*Faculty of Engineering Lab, International Islamic University Malaysia (IIUM).*
Data accessibility	***Please note:****All raw data referred to in this article must be made publicly available in a data repository prior to publication. Please indicate here where your data are hosted (the URL must be working at the time of submission and editors and reviewers must have anonymous access to the repository):* Repository name: Cognitive Load Assessment Through EEG: A Dataset from Arithmetic and Stroop Tasks Data identification number: *DOI:*10.17632/kt38js3jv7.1 Direct URL to data: https://data.mendeley.com/datasets/kt38js3jv7/1 Instructions for accessing these data: Direct download

## 1 Value of the Data

• This collection of EEG signals serves as a valuable resource for monitoring stress-induced responses in individuals engaged in diverse tasks, including the Stroop color-word test and mathematical problem-solving.
• The primary objective of this dataset is to capture stress levels induced by individual tasks, categorized into four classes representing varying degrees of mental stress (normal, low, mid, and high).
• Researchers can leverage this dataset to identify patterns in EEG signals associated with stress, offering insights into stress perception across different levels.
• In addition to EEG data, behavioral stress ratings were gathered from participants during the Stroop color-word test and arithmetic problem-solving tasks, providing a comprehensive perspective.
• These behavioral measures, included alongside the dataset, open avenues for unexplored analyses. The dataset's potential extends to clinical applications, facilitating the identification of stress in subjects.
• Furthermore, researchers can delve into source localization of EEG signals related to stress, allowing for a deeper understanding of cognitive tasks' impact on stress levels.
• Classification of EEG signals based on individual cognitive tasks enables additional inferences, and the dataset offers an opportunity to analyze which tasks elicit the highest levels of stress.

## 2 Background

Over recent years, the application of electroencephalography (EEG) in monitoring mental states has garnered significant attention, particularly within the domain of brain-computer interfaces (BCI). The investigation into EEG signal patterns as indicators of stress opens a pivotal window into the cognitive load and its implications on mental health [2]. The compilation of EEG signals, especially within the context of these tasks, offers a rich dataset for probing the neural correlates of stress. It contributes to a growing repository of data needed to develop sophisticated algorithms that can accurately classify and predict stress levels, potentially leading to advancements in both healthcare and BCI technology.

Traditional stress studies typically categorize responses as either stressed or not, missing the full range of human stress levels. Our approach divides stress into four classes: normal, low, mid, and high, capturing a more detailed spectrum of responses. This multi-class system enhances algorithm development for detecting nuanced cognitive load changes, which is essential for improving mental health interventions, learning, productivity, and human-computer interactions. This study's foundation is crucial for advancing stress classification research, with significant implications for cognitive function and well-being.

The development of this dataset is motivated by the critical need to better understand and quantify stress-related cognitive load, a common yet complex challenge in both clinical and educational settings. Despite the significant number of EEGs administered annually in clinical settings, there is a notable lack of publicly available multilevel stress data. This understanding is essential for the advancement of mental health diagnostics and therapeutic interventions, particularly in the realm of brain-computer interfaces where precise stress level detection can significantly enhance user interaction. Our dataset addresses this gap, offering a valuable resource for the research community. By recording stress levels across a spectrum (normal, low, mid, high) during tasks such as the Stroop test and arithmetic challenges, we provide a comprehensive dataset that can be used for training and validating stress detection algorithms. This contribution is expected to facilitate advancements in mental health research and the development of more nuanced, effective BCI applications.

This study's foundation is crucial for advancing stress classification research, with significant implications for cognitive function and well-being.

## 3 Data Description

This dataset is structured in one main folder (/raw_data). The /raw_data folder contains two folders

• **Arithmetic_Data:** contains 4 types of signals natural-1-natural-15, lowlevel-1- lowlevel-15, midlevel1-midlevel-15, highlevel-1 to highlevel-15.
• **Stroop_Data:** contains 4 types of signals natural-1-natural-15, lowlevel-1- lowlevel-15, midlevel1-midlevel-15, highlevel-1 to highlevel-15.

Over the past decade, the field of brain-computer interface (BCI) has experienced notable growth, witnessing developments for diverse applications such as motor imagery, prosthesis, and emotion recognition. Emotions, with their significant impact on cognition, motivation, perception, creativity, attention, learning, and decision-making, hold a crucial role in this context [3]. The visualization of mental states has shown immense potential, offering valuable insights for psychologists in diagnosing mental disorders. Notably, tasks such as the Stroop color-word test and arithmetic problem-solving have been identified as effective in eliciting mental stress responses, thereby providing an optimal setting for EEG data collection. This dataset is specifically designed to address the primary objective of capturing and quantifying the levels of stress induced in individuals during the execution of various tasks, such as the Stroop color-word test and arithmetic problem-solving. By focusing on stress levels elicited during different activities, the dataset provides a valuable resource for identifying and understanding the varying degrees of stress experienced by individuals.

### 3.1 Description of Mental Activities Tasks

The dataset is meticulously designed to evaluate stress levels induced during various cognitive tasks, with a primary focus on the Stroop color-word test and arithmetic problem-solving.

#### 3.1.1 Stroop Color-Word Test

The Stroop Color-Word Test (SCWT) serves as a well-established neuropsychological assessment tool aimed at measuring cognitive interference during the processing of diverse stimuli [4]. Leveraging its documented ability to induce stress in prior research [3], the SCWT is seamlessly integrated into our current study. Participants are assigned the task of identifying color names presented within distinct color patches and navigating through congruent and incongruent conditions. In the congruent scenario, the color name aligns with the ink color in which the word is printed, while the incongruent condition introduces a mismatch.

**Fig. 1:**
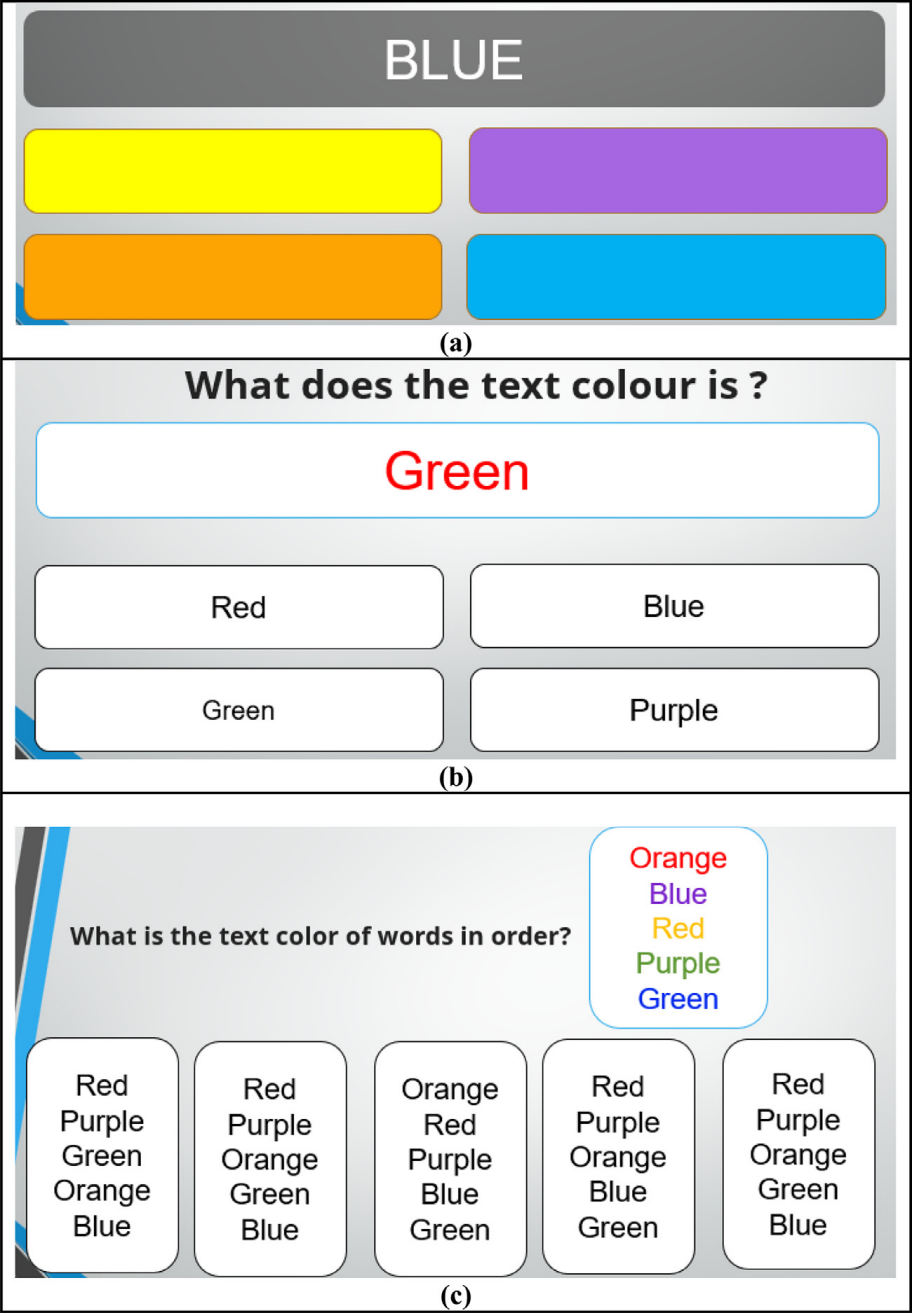
Congruent and Incongruent condition in SCWT for three different mental stress levels (a) low. (b) mid. (c) high.

As illustrated in Fig. 1, we designed three different levels of mental stress conditions using the SCWT:

Fig. 1a depicts the low-level stress condition. Here, participants are shown the word “BLUE” printed in a matching blue color. They are tasked with identifying the color name among several options, which represents a congruent scenario with minimal cognitive interference. This task is timed, requiring completion within 10 s. Fig. 1b illustrates the mid-level stress condition. In this setup, participants must identify the ink color of the word “Green,” which is printed in red. This incongruent condition creates moderate cognitive interference, increasing the participant's cognitive load. Participants are given 10 s to complete this task. Fig. 1c presents the high-level stress condition. Participants are required to identify the text color of a sequence of words, such as “Orange,” “Blue,” “Red,” “Purple,” and “Green,” presented in mismatched ink colors. This combination of congruent and incongruent elements introduces significant cognitive load and stress. This task must be completed within 20 s.

These visual representations in Fig. 1 demonstrate the varying levels of cognitive interference and stress induced by the SCWT tasks, all conducted under the pressure of a stopwatch to ensure timely completion. The progression from low to high stress conditions effectively simulates the increasing complexity and cognitive demands placed on the participants.

#### 3.1.2 Arithmetic Problem-Solving Task

The second cognitive task involves arithmetic problem-solving, a recognized stress-inducing activity [5]. In this aspect of the study, participants are tasked with mentally solving problems, mirroring the approach of the Stroop Test. The EEG data is then categorized into three distinct classes based on the varying levels of mental stress induced by different types of arithmetic problems [8].

**Fig. 2:**
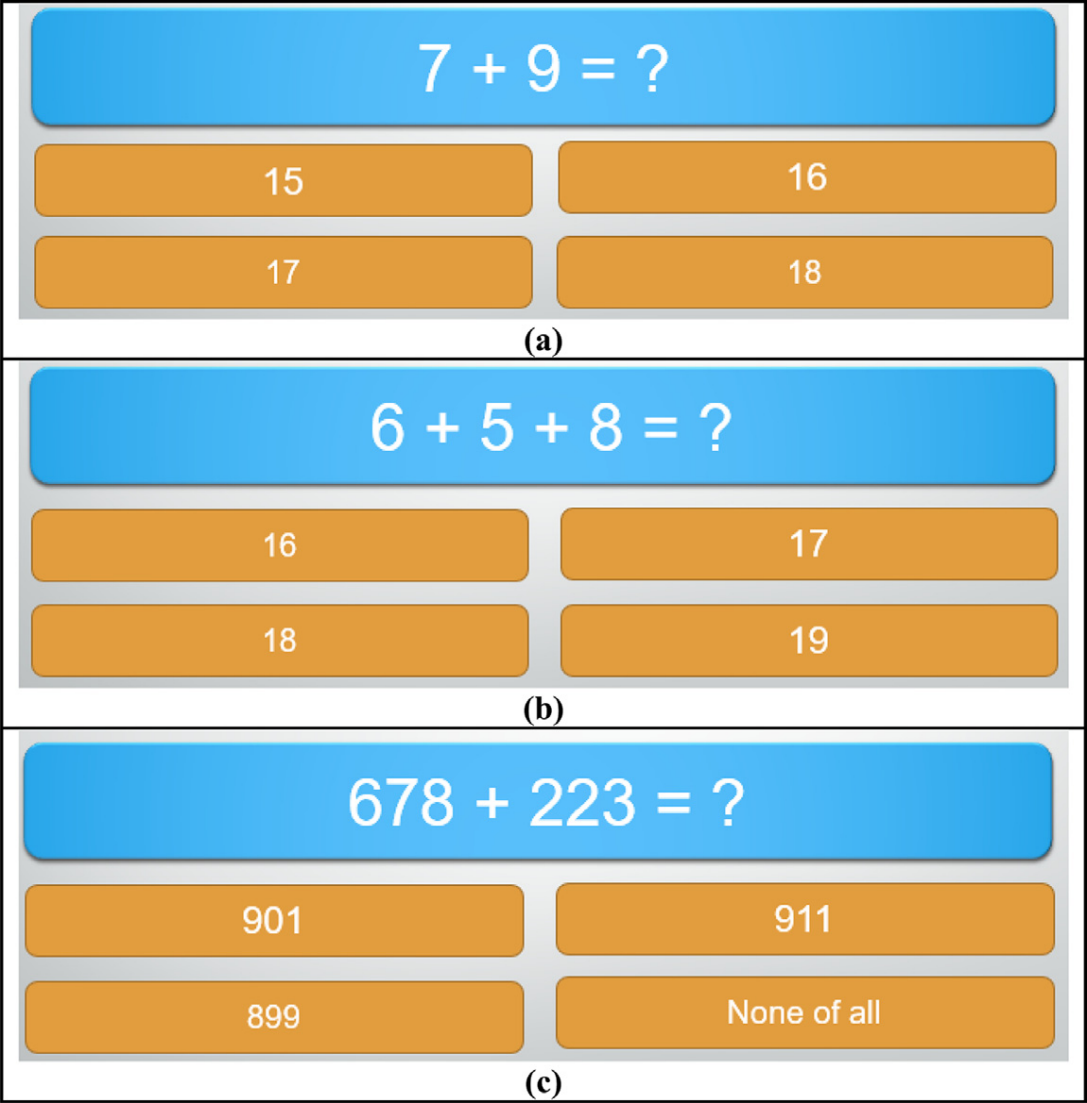
The Arithmetic Stimuli used in the proposed work at different levels in (a) low-level stress, (b) mid-level stress, and (c) high-level stress.

As illustrated in Fig. 2, we designed three different levels of mental stress conditions using arithmetic stimuli:

Fig. 2a depicts the low-level stress condition. Participants are presented with a simple arithmetic problem, such as “7 + 9 = ?”, and must choose the correct answer from a set of options (15, 16, 17, 18). This task is straightforward and induces minimal cognitive load. The task is timed, requiring completion within 10 s. Fig. 2b illustrates the mid-level stress condition. In this setup, participants solve a slightly more complex problem, like “6 + 5 + 8 = ?”, with answer choices of 16, 17, 18, 19. This task introduces moderate cognitive load and must be completed within 20 s. Whereas Fig. 2c presents the high-level stress condition. Participants are required to solve a more complex arithmetic problem, such as “678 + 223 = ?”, with multiple-choice answers (899, 901, 911, None of all). This task significantly increases cognitive load and must be completed within 20 s.

These visual representations in Fig. 2 demonstrate the varying levels of cognitive interference and stress induced by the arithmetic tasks, all conducted under the pressure of a stopwatch to ensure timely completion. The progression from low to high stress conditions effectively simulates the increasing complexity and cognitive demands placed on the participants.

Integrating these timed Stroop Color Word and arithmetic problem-solving tasks into our study allows us to systematically measure and analyze the impact of different stress levels on cognitive performance. This approach provides a comprehensive understanding of stress-related cognitive load and reinforces the robustness of our experimental design in inducing and measuring stress levels.

## 4 Experimental Design, Materials and Methods

The study was conducted on a diverse group of 15 university students, comprising 8 females and 7 males, with a mean age of 21.5 years. Participants were selected based on their health, with all individuals having no known neurological or psychiatric disorders, ensuring the reliability of EEG recordings related to cognitive stress without confounding medical factors. The population included students from various academic disciplines, providing a wide range of cognitive backgrounds.

Participants were of mixed ethnicities, reflecting a broad demographic spectrum to enhance the generalizability of the findings. None of the subjects had prior extensive experience with EEG studies or the specific tasks used in this study, which included the Stroop color-word test and arithmetic problem-solving tasks. Before data collection, all participants underwent a brief training session to familiarize themselves with the tasks, ensuring consistency in task execution.

**Fig. 3:**
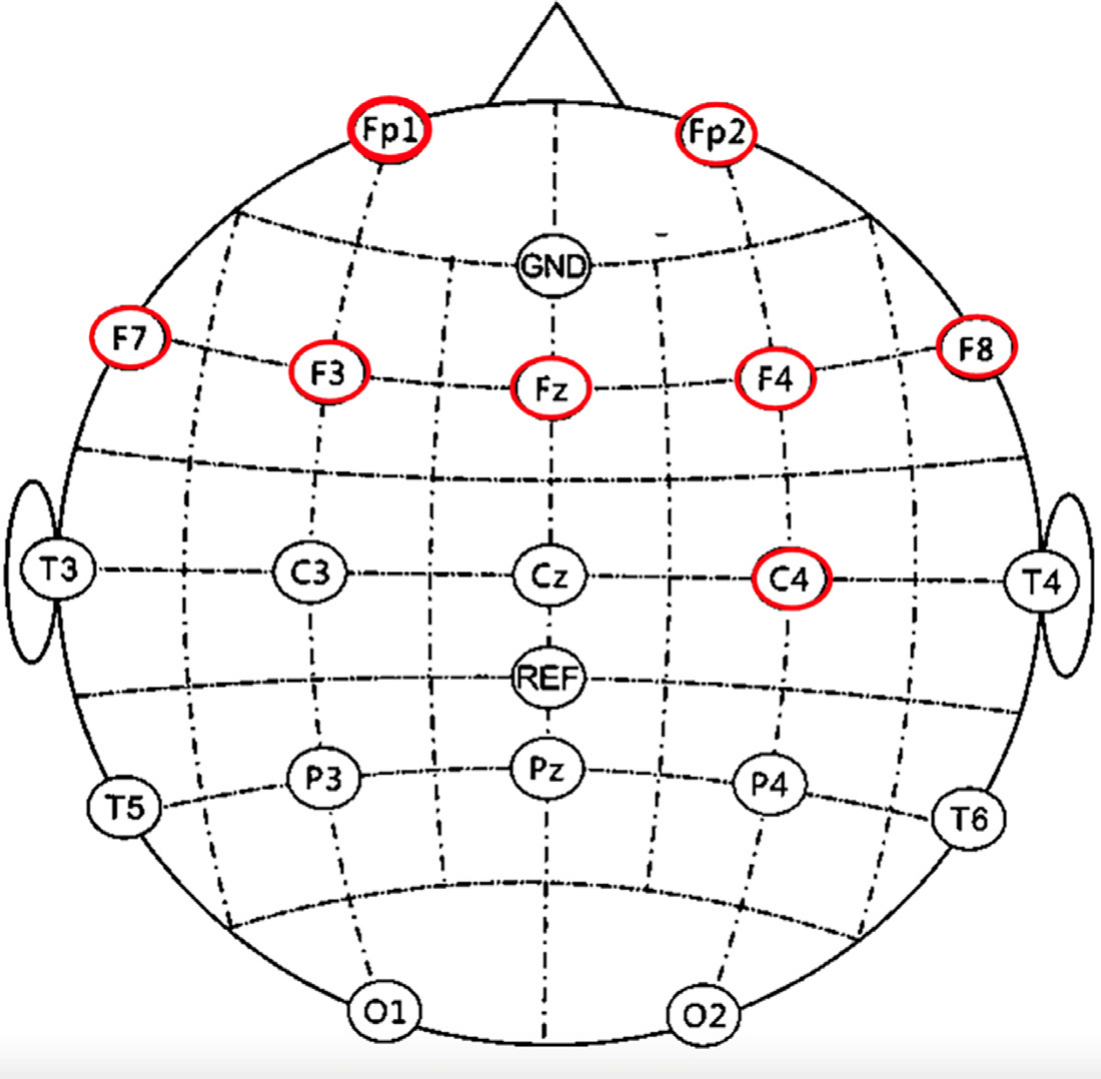
Placement of 8-channels of electrode.

This selection and characterization of the population are critical for interpreting the EEG signals in relation to cognitive load and stress levels, as they provide a controlled yet varied set of cognitive responses for analysis. The EEG data was gathered using the OpenBCI device (EEG ELECTRODECAP KIT/Coated) with Cyton board 8 channels as shown in Fig. 3. The data collection involved 8 channels Fp1, Fp2, F7, F3, FZ, F4, F8, and C4 based on the 10–20 system of electrode placement [6], targeting primarily the frontal region, a crucial area associated with cognitive functions like attention and working memory.

### 4.1 Data Recording Methodology

Mental stress has profound implications on our overall well-being, influencing everything from our cognitive abilities to our physical health. Accurately gauging mental stress can provide critical insights, paving the way for more effective interventions and support systems.

Our self-collected dataset is an endeavor to understand these intricate patterns of mental stress using EEG signals, particularly focusing on mental cognitive load.

**Fig. 4:**
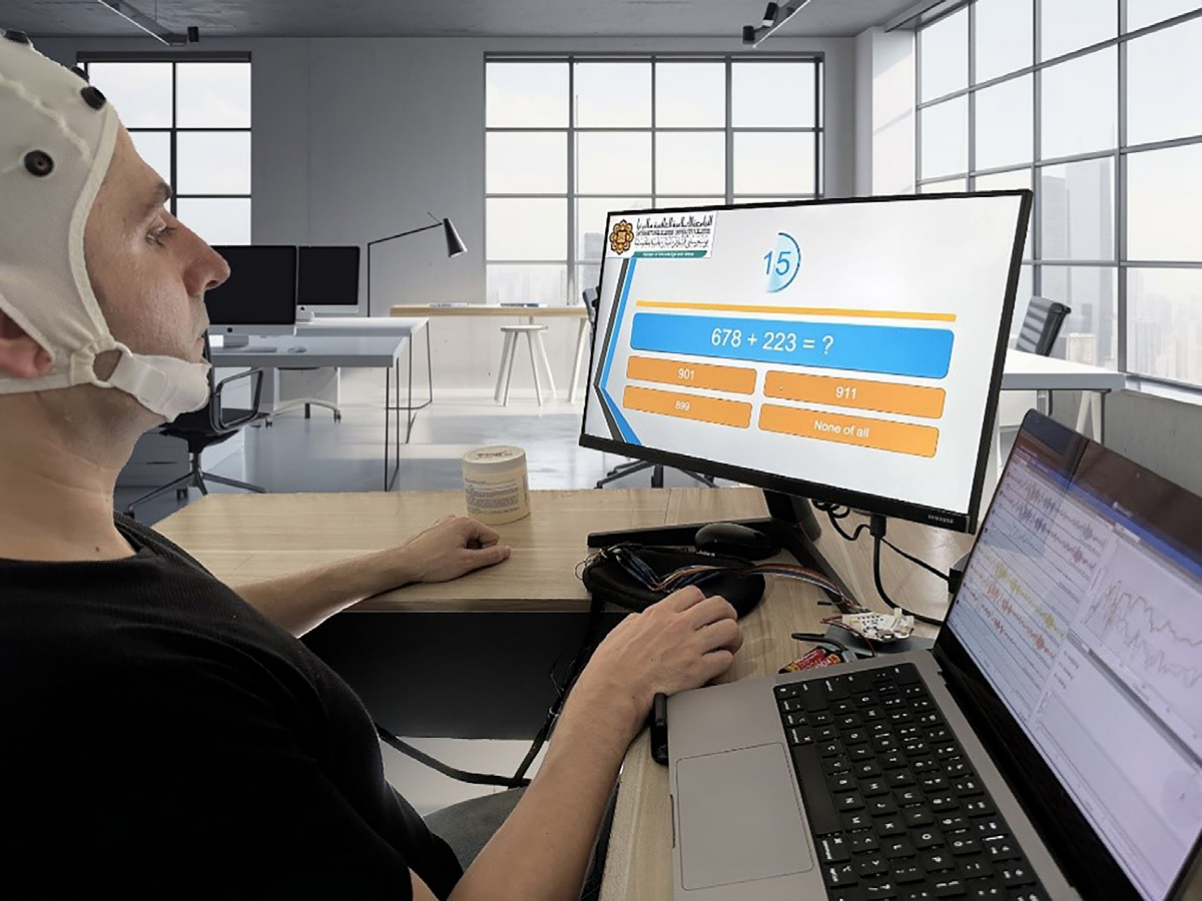
Electrode placement configuration for brain activity monitoring.

The experiment commences with the experimenters setting up the apparatus. Subsequently, the EEG device is carefully positioned on the subject's head, and clear instructions are provided to familiarize the subject with the upcoming tasks. The trial recording diagram, as depicted in Fig. 4, serves as a visual guide for the experimental setup.

Following the setup, the experimenter initiates the recording of EEG data, and the subject is directed to perform various tasks. Initially, the subject is instructed to relax for a duration of 20 s, aiming to create a baseline state of ease. Following this relaxation period, specific instructions for the Stroop color-word test are presented to the subjects, marking the commencement of the experimental tasks.

The subject is asked to perform the Stroop color-word test for 30 s. as per the following explanation:

• **Natural-level:** In this phase, the participant will be asked to relax while recording the signals for 20 s
• **Low-Level Stress:** In this phase, subjects answer six simple Stroop questions within a 10-s timeframe for each. An example is identifying the text color of the word “Blue” written in white. All responses are given by selecting color-filled boxes corresponding to the text color.
• **Mid-Level Stress**: This involves six standard Stroop questions, also with a 10-s limit per question. The complexity is slightly increased by presenting the word (e.g., “Blue”) in a color different from the text, introducing visual interference. Subjects select their answers from boxes that are colored identically to the text but are labeled with color names, adding a layer of difficulty.
• **High-Level Stress:** Subjects face a more challenging version of the Stroop task under this classification. They are presented with a set of five words in each question, each colored differently from their textual meaning (e.g., the word “Red” might be colored in blue, etc.). Within a 20-s timeframe, the question posed is to identify the actual text color of each word. Participants must then match these colors to a group of colored options provided with the question.

Following a brief relaxation period of 5 s, the subject is presented with instructions for the subsequent task lasting 10 s. During this phase, the subject is guided to engage in arithmetic problem-solving, entailing mental solutions categorized into three distinct classes. The classification is based on the varying levels of mental stress induced by diverse types of arithmetic problems. These classes are:

• **Natural-level**: In this phase, the participant will be asked to relax while recording the signals for 20 s
• **Low-Level Stress**: Subjects are given six simple arithmetic questions, with 10 s allocated for answering each. Example: Calculating the sum of two single-digit numbers, like 7 + 9.
• **Mid-Level Stress:** This involves six arithmetic questions, each incorporating three numerical values. Subjects have 20 s to respond to each question. An example question might be a combination of addition and subtraction, such as 5 + 6 - 9.
• **High-Level Stress:** In this category, subjects face six complex arithmetic questions, again with a 20-s time limit per question. These problems typically involve larger numbers. For example, a question might be 675 + 223.

**Fig. 5:**
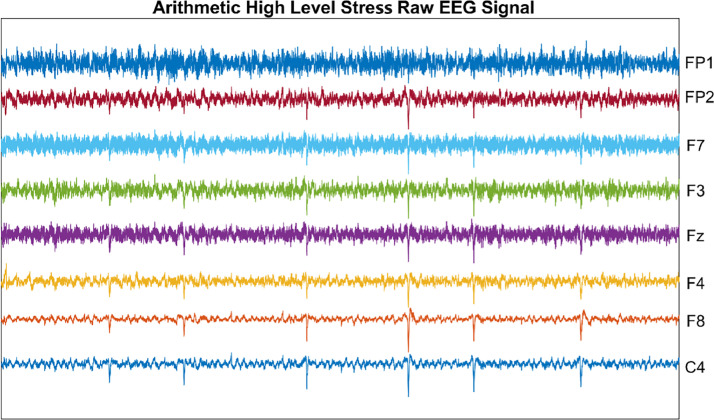
The trial recording signal.

Fig. 5 illustrates the EEG data collected during the high-level stress arithmetic task, providing insight into the neural responses elicited by cognitive stressors. The detailed visualization of these signals enables a deeper analysis of the brain's activity patterns under stress, contributing to our understanding of stress-induced cognitive load.

In summary, this study has successfully developed and characterized a multi-class EEG dataset that delineates the cognitive load and mental stress experienced by individuals. The data, derived from a balanced demographic group and obtained via a robust OpenBCI system [7], capture the complex neural responses to tasks designed to induce varying stress levels. Our findings indicate that the frontal lobe's activity, as recorded by EEG, provides significant insights into mental stress patterns.

The implications of this research are manifold. By classifying stress into distinct levels, we have provided a framework that not only advances BCI research but also enhances our understanding of stress as a multifaceted mental state. This dataset stands as a critical asset for future studies aimed at detecting early signs of serious health conditions through stress patterns, thereby facilitating timely and effective medical interventions. As we navigate the intricacies of mental health and cognitive monitoring, the value of such datasets becomes increasingly apparent. They enable the development of AI and deep learning algorithms that can interpret EEG data with unprecedented accuracy, leading to innovations in health monitoring and stress assessment. Moving forward, we anticipate that subsequent research will leverage this dataset to refine stress detection and classification techniques, explore the causal relationships between stress and health outcomes, and develop real-time adaptive systems that contribute to holistic well-being. Further studies could investigate the long-term effects of stress by integrating longitudinal data collection, thereby enhancing the predictive power of stress-related biomarkers. Additionally, there is a need to address the scalability of such systems in diverse populations and settings, ensuring that the solutions developed are accessible and effective across different cultural and socioeconomic groups. By exploring these avenues, the path forward is one of integration, where multi-disciplinary efforts converge to harness the potential of EEG data in fostering a healthier society.

## Limitations

While the dataset presents a valuable resource for stress-induced multilevel EEG signal analysis, several limitations must be acknowledged:

- Sample Diversity: The data was collected from students within a single institution, which may limit the generalizability of the findings across different demographics, cultures, and age groups.
- Behavioural Correlates: Although behavioural stress ratings were collected, they may not fully capture the subjective experience of stress, which can be influenced by numerous external and internal factors not accounted for in the study.
- Task Specificity: The dataset focuses on the Stroop color-word test and arithmetic problem-solving, which, while effective, represent only a subset of possible stress-inducing tasks. The stress levels induced by these tasks may not be entirely representative of stress in everyday life or other types of cognitive load.

## Ethics Statement

This study involving human subjects was conducted following the ethical standards outlined in the Declaration of Helsinki. Informed consent was obtained from all participants included in the study.

## CRediT authorship contribution statement

**Ali Nirabi:** Conceptualization, Data curation, Formal analysis, Investigation, Methodology, Writing – original draft, Writing – review & editing. **Faridah Abd Rahman:** Conceptualization, Methodology, Validation, Writing – original draft, Writing – review & editing. **Mohamed Hadi Habaebi:** Conceptualization, Methodology, Validation, Writing – original draft, Writing – review & editing. **Khairul Azami Sidek:** Validation, Writing – original draft, Writing – review & editing. **Siti Yusoff:** Validation, Writing – original draft, Writing – review & editing.

## Data Availability

Mendeley DataCognitive Load Assessment Through EEG: A Dataset from Arithmetic and Stroop Tasks (Original data)
